# Using the quadruple aim to understand the impact of virtual delivery of care within Ontario community health centres: a qualitative study

**DOI:** 10.3399/BJGPO.2022.0031

**Published:** 2022-10-19

**Authors:** Sara Bhatti, Simone Dahrouge, Laura Muldoon, Jennifer Rayner

**Affiliations:** 1 Alliance for Healthier Communities, Toronto, Canada; 2 Bruyère Research Institute, University of Ottawa, Ottawa, Ontario, Canada; 3 Family Physician, Somerset West CHC, Ottawa, Ontario, Canada; 4 Department of Family Medicine, University of Ottawa, Ottawa, Ontario, Canada; 5 Centre for Studies in Family Medicine, University of Western, London, Canada

**Keywords:** primary health care, virtual care, telemedicine, COVID-19, telehealth

## Abstract

**Background:**

The onset of the COVID-19 pandemic and introduction of various restrictions resulted in drastic changes to 'traditional' primary healthcare service delivery modalities.

**Aim:**

To understand the impact of virtual care on healthcare system performance within the context of Ontario community health centres (CHCs).

**Design & setting:**

Thematic analysis of qualitative interviews with 36 providers and 31 patients.

**Method:**

Semi-structured phone interviews were conducted in the autumn of 2020. Interviews were recorded, transcribed verbatim, and analysed thematically using the quadruple aim framework.

**Results:**

The transition to virtual delivery of services has had both positive and negative impacts on care. Virtual care removed certain barriers to care such as access. However, patients and providers found that phone visits impacted quality of care owing to the inability to read body language and having to rely solely on patient descriptions. Video visits allowed for a similar experience to in-person visits but technical issues constrained this interaction. Depending on the service provided, some providers felt they were not providing the same quality of care. However, providers reported reductions in no-show rates and highlighted the efficiency of virtual appointments. Providers also found they were able to collaborate at a similar level before the pandemic and saw improvements in work–life balance. Overall, patients and providers alike preferred virtual visits with those known to them, and for less complex or transactional aspects of care.

**Conclusion:**

The study described positive and negative impacts on patient care, population health, health system costs, and provider experience. These results will be useful for primary care organisations in post-pandemic planning; however, future research is needed for a deeper exploration of the impact on quality of care specifically for more complex health concerns.

## How this fits in

The COVID-19 pandemic and the introduction of various restrictions resulted in drastic changes to traditional primary healthcare service delivery modalities. However, only a handful of studies have explored how use of virtual care has impacted healthcare system performance using a standardised framework. This study explores the impact of transitioning to virtual care within the context of Ontario CHCs using the quadruple aim as a guiding framework. The findings of this article describe the impact from both the patient and provider perspective and will therefore be useful for primary care organisations in post-pandemic planning.

## Introduction

The onset of the COVID-19 pandemic and introduction of various restrictions, including government-mandated physical distancing, resulted in drastic changes to the delivery of 'traditional' primary healthcare services. This drastic shift involved moving from primarily in-person delivery to massive widespread adoption of virtual delivery of care.^1^ Recognising that this adoption will have a lasting effect on primary health care, many studies have been recently published on the experiences of patients and providers. These studies have looked at modality preferences, as well as negative and positive impacts on the quality of services delivered, including patient–provider relationships,^2,3^ quality of care,^4–7^ access to care,^8,9^ and on costs.^10^ However, very few studies have used this opportunity to understand how use of virtual care impacts healthcare system performance using the quadruple aim as a standardised framework.

The Alliance for Healthier Communities embarked on a research study to explore Ontario CHCs' experiences with rapidly transitioning to the greater use of virtual modalities (that is, phone, video, text, or email) for delivering primary health care. CHCs, which are comprehensive, salary-based primary care organisations, adhere to an evidence-informed model of care called the Model of Health and Wellbeing.^11^ This model provides a roadmap for primary healthcare delivery and is comprised of principles related to health equity, social determinants of health, ans team-based care, to name a few. The aim of this study was to understand the impact of virtual care on healthcare system performance in the context of CHCs, using the quadruple aim as a guiding framework.

## Method

### Study design

This study was a part of a larger cross-sectional study conducted to explore CHC experiences with adopting greater use of virtual care for one-on-one visits.^12^ For the qualitative aspect, a descriptive multi-case study approach was used.^13^

### Theoretical framework

**Figure 1.**
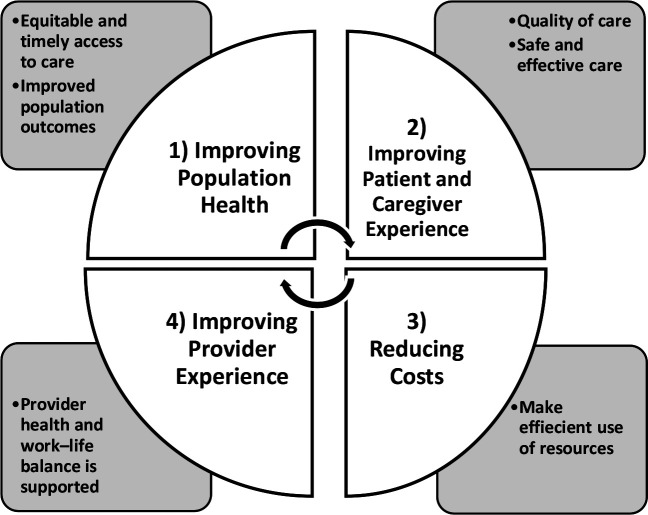
Quadruple aim framework

The quadruple aim^14–16^ is a widely accepted framework for healthcare system design consisting of the following four objectives: improving the health of populations; improving the patient and caregiver experience; reducing cost; and improving provider experience (see Figure 1). This framework was chosen as it assesses multiple domains of providing high quality care.

### Recruitment and sampling

From the larger study, 33 Ontario CHCs had volunteered to participate in the qualitative portion of the study.^12^ Of those, six were purposefully selected to maximise variation in rurality, northern geography, year established, priority populations served, and size (indicated by number of staff employed at the CHC). A convenience sampling strategy was employed to recruit interview participants through staff contact. Staff or providers contacted patients who had received care virtually and had access to a phone for the interview. Interested patients were then contacted by the first author who introduced themselves, their role at the Alliance for Healthier Communities, and the purpose of the study.

### Researcher characteristics

Research team members were of different cultural and disciplinary backgrounds; however, all had previously worked with CHCs in some capacity, either through employment or conducting research. The team included those with clinical experience working in primary care settings with diverse populations as well as varying levels of experience with conducting qualitative and health services research. Interviews were carried out by the first author, who identifies as female.

### Data collection

A semi-structured interview guide was developed to understand the experiences of primary healthcare providers (that is, primary care providers and interprofessional team members) and patients with virtual delivery of care at their respective CHC. Existing literature and the quadruple aim framework were used to design the interview guide. The guides explored perceived benefits and challenges of virtual delivery of care, impact on quality of care, and interest in virtual care post-pandemic (see appendix 1). Interviews took place during the autumn of 2020 and ran for 20–30 minutes. Interviews were conducted by the first author over the phone, recorded, and transcribed verbatim. Informed consent was obtained from all participants and all transcripts were anonymised. Interviews were conducted until no new themes had emerged.

### Data analysis

Thematic analysis was conducted in NVivo (version 12) using Morse’s four-stage approach, as outlined by Houghton *et al*.^17^ Strategies employed within each stage were guided by Miles *et al*.^18^ The first stage of analysis (comprehending) consisted of familiarising oneself with existing literature and transcripts, and initial coding using the 'broad coding' approach. Coding of transcripts was done independently by the first author and two research students. Transcripts were then compared to generate a codebook, which was maintained by the researcher. The second stage (synthesising) involved identifying patterns across cases using pattern coding. In this stage, transcripts were coded for a second round and reviewed again to ensure inter-rater agreement and credibility of analysis. This was followed by examining relationships among the data and testing them against the data in the third stage (theorising). The final stage of analysis (recontextualisation) involved comparing the findings against existing literature. Coders were in agreement for the majority of coding and disagreements regarding interpretation of codes were resolved between the coders.

## Results

### Participants

Thirty-one patients and 36 providers were interviewed (see Table 1). The majority of patients were aged ≥46 years (64%), only had phone appointments (58%), had a virtual visit with their primary care provider (77%), and had no experience with virtual care before the pandemic (84%). Providers involved in the study included primary care providers, therapists or social workers, and physiotherapists. They were primarily 31–45 years of age (50%), were employed at the centre for >5 years (61%), and had minimal experience with virtual visits (67%).

**Table 1.** Participant characteristics

	*n*	%
**Patients (*n* = 31)^a^**		
Age, years		
18–30	5	16
31–45	6	19
46–60	11	35
>61	9	29
Visit types		
Phone only	18	58
Video only	3	10
Phone and video	10	32
Provider types seen virtually		
Primary care	24	77
Therapists or social worker	8	26
Physiotherapists	5	16
Past experience with virtual care		
No experience	26	84
Minimal	5	16
**Providers (*n* = 36**)		
Age, years		
18–30	3	8
31–45	18	50
46–60	10	28
>61	5	14
Employed >5 years	22	61
Provider type		
Primary care	16	44
Therapist or social worker	16	44
Physiotherapist	4	11
Past experience with virtual care		
No experience	8	22
Minimal	28	67

^a^Some patients saw more than one provider type.

Findings from patient and provider interviews have been categorised under each dimension of the quadruple aim framework. Illustrative quotes for themes and number of transcripts referenced are highlighted in Tables 2 5 4 5.

**Table 2.** Quadruple aim 1: Improving health of populations

Sub-theme	Illustrative quote
Equitable access(*n* = 25/31 patient transcripts)(*n* = 36/36 provider transcripts)	*'My biggest barrier to health care even before the pandemic was transportation. When your appointment runs two hours late and you're sitting in a waiting room, there are better things that you could be doing with your day as opposed to having to navigate public transit.'* (London InterCommunity Health Centre, patient)*'There are days that I can’t get up and get to the centre, but now I can address those problems. I would imagine if there’s other handicapped people that would be a real asset for them too.'* (Chatham-Kent, patient)*'For one of my patients, it would cost her $20 to come here and go back home. So, of course this eliminates that cost for a lot of people.'* (Chigamik, provider)*'The limitations have much to do with poverty as anything else.'* (London InterCommunity Health Centre, provider)

**Table 3.** Quadruple aim 2: Improving patient and caregiver experience

Sub-theme	Illustrative quote
Patient–provider relationships(*n* = 25/31 patient transcripts)(*n* = 34/36 provider transcripts)	*'I wasn’t worried about my care because I knew the person that I was primarily dealing with.'* (Southeast Ottawa, patient)*'I don't think I would have done virtual care for my counselling visits. Some of the things that* [provider’s name] *and I discuss, is built on that trust. I wouldn’t have that trust with a new provider all of a sudden.'* (Chatham-Kent, patient)*'For me I prefer in person or video. I can see the person and it’s a much better conversation, and to share body language too.'* (NorWest, patient)*'For new patients, I would like having that initial appointment face to face, and then virtual. You can’t build trust and rapport very well over virtual visits, you need to be in-person for that.'* (NorWest, provider)
Care provision(*n* = 14/31 patient transcripts)(*n* = 36/36 provider transcripts)	*'* […] *I wanted to discuss one thing about my daughter’s thigh and I could only describe it with my words* […]*, so I sent a picture, but it wasn't easier to assess it.'* (Access Alliance, patient)*'A virtual appointment with video or without does not give you the complete picture that you may have in regards to having somebody in front of you. It definitely has its limitations, especially if somebody is describing something quite minimally and it’s actually quite significant.'* (Southeast Ottawa, provider)
Maintaining privacy(*n* = 24/36 provider transcripts)	*'I've explained to patients the issues of privacy and confidentiality. They’ll say, “I'm at Walmart, but I don’t care. Let’s just talk now.” I found that hard because a lot of people will say, “No, no, don't call me back.”'* (Chatham-Kent, provider)
Technical issues(*n* = 10/31 patient transcripts)(*n* = 18/36 provider transcripts)	*'* […] *there are times when the technology just doesn't cooperate and so we have to hop on a phone call instead, which can be really disruptive.'* (Chigamik, patient)*'* […] *I’m not a technical person, I’m stressed enough to make sure I do it right. Whereas, when you go to the office, you know you’re going to have your appointment no matter what.'* (NorWest, patient)*'It’s very frustrating for us because we get behind and we're spending time coaching the patient about how to use the technology. Not a good use of our time.'* (London InterCommunity Health Centre, provider)
Aspects of care suited for virtual delivery(*n* = 31/31 patient transcripts)(*n* = 36/36 provider transcripts)	*'I do like the in-person scenario. However, there are things that come up from time to time that are easy for me to deal with by phone.'* (Chigamik, patient)*'Yeah, I mean* [virtual appointments] *are so much easier. I probably benefit more from my therapy sessions because I'm more relaxed at home and so I'm able to be more present because I didn’t have all of that stress of getting there.'* (London InterCommunity Health Centre, patient)*'I think that virtual care is excellent in certain ways* […] *I would often question why I'm bringing an 85-year-old woman out of her home in February to come talk about her blood work or her bone density test?'* (Chigamik, provider)*'* […] *personally, I prefer in person. That’s always better for counselling. You need to understand the person, the story, how they feel, how they lived through their issues, and all the clues are important that you see in person.'* (Chatham-Kent, provider)

**Table 4.** Quadruple aim 3: reducing costs

Sub-theme	Illustrative quote
Greater efficiency(*n* = 33/36 provider transcripts)	*'I have a lot fewer no-shows because I can catch them with the phone or with video. This has removed barriers so patients can actually come to counselling where if they were coming physically, they're not going to show up.'* (Chigamik, provider)*'Usually, I give them a call, sometimes if they don’t answer right away, I call them again ten minutes later or try a different number. In-person, I wouldn’t be able to do, it’s either they show up or they don’t'.* (NorWest, provider)

**Table 5.** Quadruple aim 4: Improving provider experience

Sub-theme	Illustrative quote
Team communication(*n* = 22/36 provider transcripts)	*'* […] *management started a COVID café, which was not only a chance to learn different things relevant for your job, but also to connect with other staff members and feel less isolated.'* (Southeast Ottawa, provider)*'The system that we've got is so user-friendly to be able to stay connected with my colleagues. No, it’s still just as good as ever.'* (London InterCommunity Health Centre, provider)
Work–life balance(*n* = 9/36 provider transcripts)	' […] *it’s been amazing to have more time in the morning. You don't spend all that time getting ready, you can take a walk, and enjoy the sunshine with your coffee outside. When your workday ends at four, it really ends, there’s no commute, so those are definitely a personal benefit.'* (Southeast Ottawa, provider)
Additional training(*n* = 12/36 provider transcripts)	*'There was no training on how to adapt your delivery of care virtually or even how to use maybe the PS Suites video platform.*' (NorWest, provider)*'I think the only hindrance was technological. The fact that we were all — I don’t think many of us had used Zoom before. There was a learning curve there.*' (Access Alliance, provider)

### Quadruple aim 1: Improving health of populations

#### Equitable access

Patients and providers alike reported lower costs to patients as a result of transitioning to virtual visits, in particular for those patients who needed to pay for transportation or parking, arrange childcare, or work fixed schedules and are required to take time off for visits. Patients with mobility or chronic health conditions that made travelling physically difficult and costly also found virtual visits more accessible overall.

However, providers highlighted that virtual visits were less accessible for individuals living in poverty, newcomers, and those who are experiencing homelessness owing to limited or no access to technology. Older patients, specifically, reported issues with digital literacy and patients from rural areas were further impacted by poor internet connection. According to providers, those at risk of domestic violence and individuals who lived in shared homes had difficulty participating in virtual visits owing to privacy and confidentiality limitations.

### Quadruple aim 2: Improving patient and caregiver experience

#### Patient–provider relationships

An important consideration for both providers and patients was having an established relationship before participating in a virtual visit, regardless of modality. Generally, providers felt they could provide the same quality of care as in-person visits for patients known to them. Similarly, patients preferred virtual visits with providers with whom they had a trusting relationship. In fact, over two-thirds of patients noted the importance of that historical relationship in contributing to their willingness and comfort in receiving care virtually.

During telephone visits, providers found their older patients were less likely to discuss their concerns compared with when seeing them in person. They described difficulty connecting with these clients and providing reassurance as a potential explanation. Older patients, who had received care over phone visits, also found it harder to connect to their regular provider(s) and described phone visits as impersonal. Video appointments were preferred for these reasons by both providers and patients.

#### Care provision

In comparison with video visits, providers felt that phone visits had impacted their ability to provide quality care. This was owing to relying purely on their patients' descriptions and being unable to observe patients' reactions to the information being discussed. These concerns were also cited by patients. Dieticians and therapists emphasised not being able to deliver the same quality of care owing to the inability to use educational materials. Some were able to mitigate this by emailing resources; however, this was dependent on the patient’s digital literacy. Some patients found counselling visits less therapeutic as they were uncomfortable discussing their concerns in a virtual space. Alternatively, patients with social anxiety described feeling more at ease when receiving care in the comfort of their home.

#### Maintaining privacy

Providers suggested that patients seemed less concerned about their privacy especially during phone visits, as they would sometimes answer while busy with other tasks or with others present. This also frequently resulted in patients becoming distracted during visits. In some cases, patients were unable to maintain privacy owing to their living situations, although in other instances, providers suggested that patients may view these visits as less formal.

#### Technical issues

The most common technical issue mentioned was unstable internet connection during video visits, often leading to poor video and audio quality. Although patients preferred video visits, some opted for phone owing to this issue and because they found switching modalities when video did not work disruptive to their care. Providers found video visits challenging when they had to assist patients in resolving technical issues, leaving less time for the actual appointment. Patients with limited digital literacy expressed feelings of frustration when setting up their video visits and trying to minimise technical issues on their end. Providers also found it challenging to use their personal phones for phone visits, which required blocking their numbers, resulting in patients not answering and being unable to return calls.

#### Aspects of care suited for virtual delivery

Patients and providers alike were in agreement that virtual visits were suitable for follow-up calls, medication reviews, and prescription renewals. Both also felt that virtual visits were convenient for those with chronic health conditions if they were familiar enough with their condition. In regard to counselling, both preferred virtual visits for mild cases of anxiety or depression. Physiotherapists were happy to offer virtual visits after the initial visit, so they could fully assess their patient’s situation and have a better picture of their concerns. Patients found that virtual physiotherapy visits worked better when they were performing exercises they had done before.

### Quadruple aim 3: Reducing costs

#### Improving efficiency

Almost half of all providers reported improvements in their no-show rates. Reasons cited included stay-at-home orders, removing access-related barriers for patients, and the ability to reach patients even if they had forgotten their appointment. Providers were also able to follow-up with other patients during missed or cancelled visit slots. One-third of all providers found they had an easier time keeping visits on time as they did not have to wait for the patient to walk in or to pack up their belonging when leaving. Generally, providers found it more time-efficient to conduct follow-up visits virtually.

### Quadruple aim 4: Improving provider experience

#### Team communication

Providers used the instant messaging feature of CHC’s electronic medical record (EMR) system to maintain a similar level of communication as before the pandemic. Overall, providers felt that they were generally able to collaborate with their peers when providing care, and that only the method of communication had changed. Some providers also mentioned the benefit of their managers setting up frequent meetings that allowed staff to connect and discuss issues they encountered during the transition.

#### Work–life balance

One-quarter of providers, primarily from urban centres, had expressed the benefits of having extra time in their day owing to not having to commute to work during the week.

#### Additional training

Some providers commented that having additional training for navigating virtual platforms, as well as adapting care for virtual delivery, would have better prepared them when conducting video visits. Physiotherapists and providers offering counselling services, in particular, highlighted the challenge of adapting care provision for virtual delivery and had concerns around treatment effectiveness.

## Discussion

### Summary

The study sought to explore the impact of transitioning to greater use of virtual care on healthcare system performance within the context of CHCs. Details around the processes involved in this transition have been described elsewhere.^12^ Using the theoretical framework, both positive and negative impacts were found on population health, patient experience, health system costs, and provider experience. The transition to virtual delivery of care had removed access barriers, including cost and transportation, but the need for technology created new barriers. Concerning patient experience, provider relationships were easily maintained through virtual visits with the exception of older patients. Quality of care, however, was impacted during phone visits when being physically present was required for adequate care provision. Video visits allowed for a similar experience to in-person visits; however, technical issues constrained this interaction. Despite these challenges, patients and providers alike voiced a preference for continuing the option for virtual visits for specific aspects of their care.

Providers revealed that they had a harder time providing care for older patients and patients who were new to the practice. Depending on the service provided, some also did not feel that they were providing the same quality of care owing to limitations of phone visits, challenges in maintaining privacy, and technical issues. In this study, the quadruple aim of reducing costs was related to reported reductions in no-show rates and cancelled visits, utilising missed or cancelled visits to follow-up with other clients, and virtual appointments being more time-efficient than in person visits. With respect to provider experience, the study found providers were able to collaborate with peers at a similar level before the pandemic and saw improvements in work–life balance. However, additional training would have been beneficial in helping providers adapt care provision for virtual delivery.

### Strengths and limitations

Interviewing both providers and patients was a strength of this study as it provided a dual perspective on the rapid transition to virtual delivery of care. However, because the sample of patient participants was recruited using a convenience sampling strategy, the sample potentially favoured patients with positive experiences. In addition, the patient sample did not include those who were unable to access care virtually, further limiting the generalisability of the findings. The study also did not include any provider or patient characteristics when citing quotes to protect their confidentiality.

### Comparison with existing literature

Although very few studies have used the quadruple aim framework to understand the impact of virtual care during the COVID-19 pandemic, many of the findings have been reflected in other recently published literature. Studies have noted poor internet connection, absence of physical examination,^4–7^ and loss of human connection and social contact^7,18^ as negatively impacting patients' experiences with virtual care. Other research has described impacts on patient-provider relationships owing to lack of body language, confidentiality concerns, and technical issues.^3,19,20^ Two studies in particular similarly highlighted the importance of having an established and trusting patient–provider relationship for successful virtual visits.^2,6^

Other studies identified similar specific aspects of care that may be better suitable for virtual delivery. For example, issues that do not require physical assessment, follow-up test results, counselling, discussing treatment options,^6,21,22^ and management of chronic diseases.^23^ Comparable with the present study, mental health care was described as the least desirable for virtual modalities,^22^ and in one study there was a split with some patients preferring in-person visits, as those felt more therapeutic, and others preferring phone visits, as those felt more comfortable.^2^

Like several other studies, the present study found that virtual care delivery eliminated certain barriers, such as costs and transportation, and increased access to care.^2–6,23–27^ Removing such barriers can lead to more equitable access and contribute to better population health; however, technology requirements also creates new barriers, which should not be ignored. Studies have shown disparities between different populations accessing care virtually and have reported on the challenge of providing care to those with lower incomes or experiencing homelessness.^8,27,28^ Moving forward, ensuring equitable access will be essential for greater adoption of virtual modalities.^19,21,25,29–31^

In regard to reducing health costs, one study similarly noted improvements in providers' no show rates but did not elaborate further.^5^ Another study evaluating a telemedicine programme found no changes in its no-show rates; however, it did report increased efficiency of care as video visits were shorter than in-person visits.^32^

A key aspect of the quadruple aim framework is improving provider experience and in the present study providers cited improvement in work–life balance; however, this was only cited by one other study.^5^ Providers within the present study did not experience common challenges other providers had during the rapid transition, such as reimbursement and billing issues,^33^ and poor integration of virtual care technologies within EMR systems.^5^ This was a direct result of CHC’s salary-based funding model in addition to their EMR system having video capabilities embedded within. However, providers in one study similarly reported that although virtual platforms were relatively easy to use, they did not have the opportunity to become comfortable with the platform before using it, and formal training would have helped providers optimise the platform for virtual delivery. In the same study, providers highlighted that adapting interventions that required a physical examination was difficult and consequently many were concerned about diagnosis and treatment effectiveness.^34^ Given that some primary health care will continue to be offered and delivered virtually post-pandemic, dedicated training is essential for ensuring providers feel confident in providing care virtually.

### Implications for research and practice

In this study, the quadruple aim framework was used to explore the impact of using virtual delivery of care within Ontario CHCs. The study described both positive and negative impacts on patient experience, population health, health system costs, and provider experience. These results will be useful for primary care organisations in post-pandemic planning; however, future research is needed for a deeper exploration of the impact on quality of care for complex health concerns.

## Data

The dataset relied on in this article is available from the corresponding author on reasonable request.
